# Comparison of Single-Freezing Temperature-Guided and Conventional Time-to-Isolation-Guided Protocols of Cryoballoon in Atrial Fibrillation Ablation

**DOI:** 10.14740/cr2202

**Published:** 2026-06-05

**Authors:** Xiao Yu Liu, Jie Zheng, Ku Lin Li, Hua Yan You, Shi Peng Dang, Yu Fei Dai, Hai Wei Geng, Yun Lai Gao, Ru Xing Wang

**Affiliations:** a Department of Cardiology, Wuxi People’s Hospital Affiliated to Nanjing Medical University, Wuxi, Jiangsu 214023, China

**Keywords:** Cryoballoon, Ablation, Atrial fibrillation, Time-to-isolation, Temperature

## Abstract

**Background:**

The study aimed to compare the efficacy and safety of simplified single-freezing protocol with achieving −40 °C within 60 s of freezing as the observation index and the conventional time-to-isolation (TTI)-guided double-freezing protocol using second-generation cryoballoon in the treatment of atrial fibrillation (AF).

**Methods:**

A retrospective analysis was performed. After propensity score matching, 146 patients who underwent conventional TTI-guided protocol freezing (conventional group) and 146 patients who underwent single-freezing temperature-guided protocol freezing (simplified group) using second-generation cryoballoon were included. Procedure time, X-ray time and dose, complications, success rate, and recurrence rate during follow-up were analyzed.

**Results:**

Compared with the conventional group, the simplified group showed a significant reduction in procedure time (98.8 ± 16.3 vs. 79.4 ± 12.7 min, P < 0.001), and X-ray time (24.3 ± 6.8 vs. 17.8 ± 4.9 min, P < 0.001) and dose (681.1 ± 337.8 vs. 540.1 ± 343.7 mGy, P < 0.001). There was no significant difference in the success rate of acute pulmonary vein isolation between the two groups of patients (145/146 vs. 146/146, P = 0.975) and the proportion of maintaining sinus rhythm during follow-up (114/146 vs. 109/146, P = 0.842). Cox regression analysis showed that simplified protocol is not a predictive factor for recurrence in AF cryoablation.

**Conclusions:**

In summary, the single-freezing temperature-guided protocol demonstrates comparable efficacy and safety to conventional TTI-guided protocol of second-generation cryoballoon in the AF ablation with the advantages of reducing procedure time and radiation exposure.

## Introduction

Cryoablation therapy for atrial fibrillation (AF) has become a routine treatment, with a success rate comparable to radiofrequency ablation [1, 2]. However, the optimal cryoablation parameters required to achieve comprehensive, persistent, and transmural lesions are still unclear [3]. Most studies recommend freezing strategies that emphasize time-to-isolation (TTI) < 60 s, which is considered a reliable threshold for predicting persistent pulmonary vein (PV) isolation [4]. However, visualization of TTI is not always possible. Some researchers suggest that a temperature of −40 °C at 60 s can be used as a predictive indicator for sustained PV isolation without requiring TTI [5, 6]. Nevertheless, few studies have investigated this approach, particularly in non-paroxysmal AF cases, and comparative data with conventional TTI-guided double-freezing protocols are lacking.

Since 2019, our center has been using second-generation cryoballoons for AF treatment, following the conventional cryoablation protocol recommended by the Chinese consensus. With the widespread using of second-generation balloons which offer higher freezing efficiency [7], clinicians have greater flexibility in determining freezing duration and frequency [8]. In 2022, our center gave up conventional protocol and choose the single-freezing temperature-guided protocol based on prior experience. Therefore, this study aimed to compare the efficacy and safety of single-freezing temperature-guided protocol and double-freezing TTI-guided protocol using second-generation cryoballoon in the AF ablation.

## Materials and Methods

### Study population

A retrospective analysis was performed on patients with paroxysmal AF who underwent cryoablation therapy in the Department of Cardiology at Wuxi People’s Hospital from January 2020 to May 2024. After excluding patients aged < 18 or > 75 years, and those with concomitant obstructive or diffuse pulmonary dysfunction and congenital heart disease, 153 patients treated with conventional protocol (conventional group) and 318 patients treated with simplified protocol (simplified group) were included in this study.

### Routine procedure section

Puncture the left femoral vein, and implant coronary sinus electrodes and right ventricular electrodes. Perform atrial septal puncture by the long sheath through right femoral vein, and then perform left atrial (LA) and PV angiography. Exchange the long sheath for a Flex catheter, and insert the Achieve mapping catheter and second-generation balloon into the left atrium.

### Conventional procedure section

According to the 2019 consensus recommendation [9], adjust the Achieve to the PV and attempt to record the PV potential. If the PV potentials are detectable, the relative position between the cryoballoon and the PV opening should be adjusted simultaneously. Single PV angiography should be used to verify complete occlusion of the target PV opening, and the “proximal seal technique” should be applied to bring the cryoballoon closer to the vestibular position of the PV before performing freezing. At the same time, TTI from the start of cryoablation to the disappearance of PV potential should be recorded. If TTI < 60 s, the first freezing should take 180 s, and the second consolidation freezing should take 120 s. If TTI > 60 s, freezing should be stopped and the cryoballoon position should be readjusted to achieve better occlusion. If TTI cannot be recorded, with good balloon occlusion, perform the first freezing for 120 s, and repeat the freezing after rewarming for 180 s. During the freezing period, maintain a temperature of −55 to −35 °C until all PV potentials disappear and observe for 20 min. Use Achieve electrodes to check the PVs one by one to verify if the PV potential has recovered. If there is any recovery, perform additional freezing until the PV electrical isolation is completed. In addition, persistent AF increases LA posterior wall freezing.

### Simplified procedure section

Adjust the Achieve to attempt recording PV potential. If the potential can be recorded, mark the Achieve position with X-rays. Then, insert the Achieve deep into the PV and adjust cryoballoon as conventional procedure section for complete occlusion. Observe whether the temperature drops to −40 °C within 60 s after freezing. If the temperature drops to −40 °C within 60 s, the freezing process ends after 180 s. If the temperature reaches −40 °C within 60–120 s, freeze once for 240 s. Otherwise, stop freezing and readjust the cryoballoon position to achieve better occlusion. The remaining steps were identical to the conventional protocol group.

### Follow-up

Follow-up was performed for all patients in outpatient clinic at 1, 3, 6, and 12 months, respectively after procedures. Holter monitoring was routinely examined when patients experienced palpitations after cryoablation in order to obtain the patients’ clinical manifestations and the occurrence of arrhythmias. The first 3 months after the procedure are the blank period, and those who have a documented episode of AF or atrial tachycardias lasting > 30 s from 3 months after procedure to the end of follow-up were defined as AF recurrence.

### Record and explanation for the parameters related to the procedure

Normal PV anatomy is defined as the presence of two PVs on each side. PV variation is defined as the presence of more than two PVs on one side and/or a common trunk of PVs. Major complications include acute myocardial infarction, stroke, major bleeding, severe PV stenosis, pericardial tamponade, LA–esophageal fistula, and permanent phrenic nerve injury. Minor complications include hematoma at the puncture site, pericarditis, subcutaneous hemorrhage, and transient phrenic nerve palsy. Success of first anatomical attempt is defined as the absence of PV potentials after the first ablation circle, and no recovery of potentials is observed during the check. Acute success is defined as achievement of entrance block confirmed after the final freeze application, with no potentials recorded in any PV at the end of the procedure.

### Statistical analysis

Quantitative data are presented as mean ± standard deviation (SD) and analyzed using SPSS 26.0. Categorical variables are expressed as ratios and percentages. Normally distributed data were compared using the Student’s *t*-test between the two groups. Mann–Whitney test was used, if the two groups of data had a non-normal distribution. The χ^2^ or fisher test was used to measure the association for categorical variables. A Kaplan–Meier analysis was used to compare the probability of AF recurrence in two groups and differences between groups were assessed using the log-rank test. Multivariate analysis was performed using Cox regression test to identify the risk factors of AF recurrence after procedure. To minimize selection bias due to the retrospective study design and baseline imbalances, propensity score matching (PSM) was performed. Patients in the conventional group were matched 1:1 to those in the simplified group using the nearest-neighbor matching algorithm with a caliper of 0.2 SDs of the propensity score. P < 0.05 indicates a statistically significant difference.

### Ethical statements

This retrospective study was conducted in accordance with the principles of the Declaration of Helsinki and was approved by the Research Ethics Committee of Wuxi People’s Hospital (Approval No. KY25110).

## Results

### Comparison of patients’ baseline characteristics

The general comparison of the two groups of patients and the results of PSM are shown in Table 1. There was no statistically significant difference in gender, age, height, weight, body mass index, proportion of underlying diseases, left ventricular ejection fraction, LA diameter, course of AF, and CHA_2_DS_2_-VASc score between the two groups (P > 0.05).

**Table 1:** Comparison of the Overall Procedural Parameters of Two Groups

	Conventional group (n = 153)	Simplified group (n = 318)	P value	PSM conventional group (n = 146)	PSM simplified group (n = 146)	P value
Gender (male/female)	92/61	190/128	0.937	87/59	86/60	0.905
Age (years)	61.1 ± 11.1	61.8 ± 9.8	0.480	61.1 ± 11.3	60.8 ± 10.2	0.778
Height (cm)	167.1 ± 8.4	167.4 ± 8.3	0.761	167.1 ± 8.3	166.5 ± 8.0	0.576
Weight (kg)	69.5 ± 10.7	70.8 ± 11.1	0.234	69.7 ± 10.7	69.3 ± 11.0	0.732
BMI (kg/m^2^)	24.8 ± 2.9	25.2 ± 3.1	0.190	24.9 ± 2.9	24.9 ± 3.1	0.975
Course of AF (months)	48.7 ± 69.9	47.6 ± 56.4	0.190	49.7 ± 71.1	50.1 ± 63.5	0.382
Paroxysmal AF	94 (61.4%)	189 (59.4%)	0.677	89 (61.0%)	85 (58.2%)	0.633
Basic diseases						
Hypertension	86 (56.2%)	201 (63.2%)	0.145	85 (58.2%)	84 (57.5%)	0.905
CHD	19 (12.4%)	38 (11.9%)	0.884	19 (13.0%)	17 (11.6%)	0.722
Diabetes	20 (13.1%)	57 (17.9%)	0.182	20 (13.7%)	18 (12.3%)	0.728
Stock/TIA	12 (7.8%)	33 (10.4%)	0.381	12 (8.2%)	12 (8.2%)	1.000
Heart failure	5 (3.3%)	4 (1.3%)	0.136	1 (0.7%)	2 (1.4%)	1.000
LA diameter (mm)	40.9 ± 5.6	40.6 ± 5.8	0.627	40.7 ± 5.5	40.4 ± 6.2	0.734
LVEF (%)	62.5 ± 3.6	62.9 ± 3.9	0.291	62.6 ± 3.6	62.9 ± 4.2	0.502
CHA_2_DS_2_-VASc	1.4 ± 1.0	1.5 ± 1.1	0.254	1.4 ± 1.0	1.4 ± 1.1	0.801
Antiarrhythmic drugs						
β-blocker	139 (90.8%)	300 (94.3%)	0.159	134 (91.8%)	140 (95.9%)	0.144
Amiodarone	127 (83.0%)	255 (80.2%)	0.464	123 (84.2%)	117 (80.1%)	0.359
Propafenone	17 (11.1%)	36 (11.3%)	0.946	15 (10.3%)	16 (11.0%)	0.849

AF: atrial fibrillation; BMI: body mass index; CHD: coronary atherosclerotic heart disease; LA: left atrial; LVEF: left ventricular ejection fraction; PSM: propensity score matching; TIA: transient ischemic attack.

### Procedure related parameters

The comparison of procedure-related parameters between the two groups of patients is shown in Table 2. Compared with the conventional group, the simplified group showed a significant reduction in procedure time (98.7 ± 15.8 vs. 82.3 ± 12.5, P < 0.001), and X-ray time (24.2 ± 6.8 vs. 18.8 ± 5.4 min, P < 0.001) and dose (676.4 ± 333.0 vs. 539.6 ± 387.3 mGy, P < 0.001). The procedure time, and X-ray time and dose for routine and freezing procedures, were separately counted. It was found that the shortened procedure time, and X-ray time and dose in the simplified group all occurred during the cryoablation operation stage. Compared with the conventional group, the applications and duration of PV freezing were significantly reduced in the simplified group.

**Table 2:** Procedural Parameters

	PSM conventional group (n = 146)	PSM simplified group (n = 146)	P value
Procedure time (min)	98.7 ± 15.8	82.3 ± 12.5	< 0.001
Routine procedure time (min)	36.8 ± 5.4	37.9 ± 6.5	0.125
Freezing procedure time (min)	61.8 ± 16.4	44.4 ± 12.9	< 0.001
Total X-ray time (min)	24.2 ± 6.8	18.8 ± 5.4	< 0.001
Routine procedure X-ray time (min)	7.7 ± 2.0	7.7 ± 1.9	0.854
Freezing procedure X-ray time (min)	16.5 ± 6.3	11.1 ± 5.2	< 0.001
Total X-ray dose (mGy)	676.4 ± 333.0	539.6 ± 387.3	< 0.001
Routine procedure X-ray dose (mGy)	160.4 ± 70.6	177.9 ± 84.3	0.098
Freezing procedure X-ray dose (mGy)	516.0 ± 281.3	361.7 ± 341.7	< 0.001
PV variant	26 (17.8%)	27 (18.5%)	0.879
Total freeze applications	9.3 ± 1.6	4.9 ± 1.3	< 0.001
Total freeze duration (s)	1,279.1 ± 158.7	824.4 ± 116.1	< 0.001
LSPV	130 (89.0%)	129 (88.4%)	0.853
LSPV freeze applications	2.2 ± 0.5	1.2 ± 0.5	< 0.001
LSPV freeze duration (s)	311.5 ± 27.8	199.0 ± 34.1	< 0.001
LSPV nadir temperature (°C)	−50.8 ± 4.4	−50.0 ± 5.1	0.166
LIPV	130 (89.0%)	129 (88.4%)	0.853
LIPV freeze applications	2.3 ± 0.6	1.2 ± 0.5	< 0.001
LIPV freeze duration (s)	316.9 ± 39.1	203.6 ± 45.7	< 0.001
LIPV nadir temperature (°C)	−49.1 ± 5.5	−49.2 ± 5.8	0.615
RSPV	146 (100%)	146 (100%)	-
RSPV freeze applications	2.2 ± 0.6	1.3 ± 0.5	< 0.001
RSPV freeze duration (s)	315.3 ± 42.4	215.0 ± 51.3	< 0.001
RSPV nadir temperature (°C)	−49.5 ± 5.8	−48.9 ± 6.5	0.393
RIPV	146 (100%)	146 (100%)	-
RIPV freeze applications	2.6 ± 0.8	1.2 ± 0.6	< 0.001
RIPV freeze duration (s)	341.4 ± 56.9	211.1 ± 47.5	< 0.001
RIPV nadir temperature (°C)	−43.9 ± 6.3	−44.9 ± 5.9	0.110
LCPV	16 (11.0%)	17 (11.6%)	0.853
LCPV freeze applications	2.8 ± 0.9	1.8 ± 1.1	0.007
LCPV freeze duration (s)	363.8 ± 86.2	285.9 ± 107.2	0.009
LCPV nadir temperature (°C)	−41.3 ± 4.4	−38.3 ± 4.8	0.071
RMPV	10 (6.8%)	10 (6.8%)	1.000
RMPV freeze applications	2.4 ± 0.5	1.2 ± 0.4	< 0.001
RMPV freeze duration (s)	336.0 ± 26.3	210.0 ± 42.4	< 0.001
RMPV nadir temperature (°C)	−46.9 ± 4.1	−45.9 ± 5.6	0.319

LCPV: left common pulmonary vein; LIPV: left inferior pulmonary vein; LSPV: left superior pulmonary vein; PSM: propensity score matching; PV: pulmonary vein; RIPV: right superior pulmonary vein; RMPV: right middle pulmonary vein; RSPV: right inferior pulmonary vein.

### Follow-up

**Figure 1:**
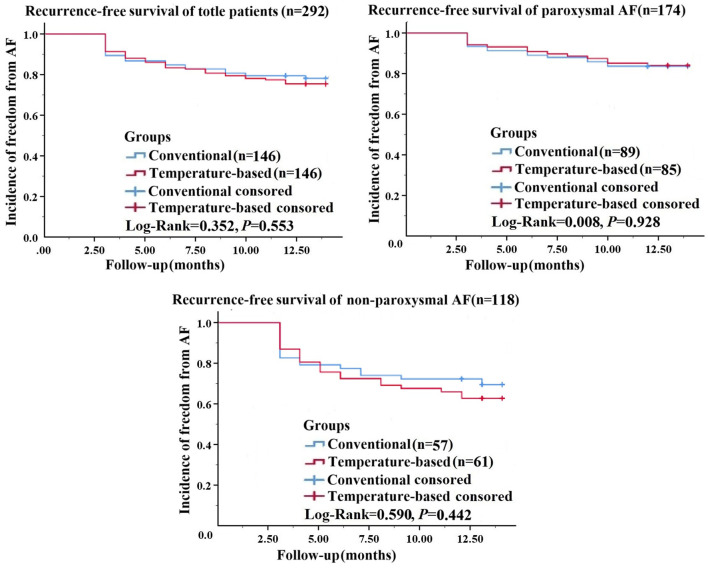
Kaplan–Meier survival analysis of patients with sinus rhythm maintenance after procedure in the conventional time-to-isolation-guided protocol (conventional group) and single-freezing temperature-guided protocol (simplified group).

The comparison of outcomes of procedure is shown in Table 3 and Figure 1. Compared with the conventional group, the success rate of the first anatomical attempt (without the need for additional freezing during the same procedure, 143/146 vs. 132/146, P = 0.006) was lower in simplified group. However, the acute success rate (145/146 vs. 146/146, P = 1.000) had no significant difference. The rate of maintaining sinus rhythm during follow-up (114/146 vs. 109/146, P = 0.842) was similar in the two protocols, both in paroxysmal and non-paroxysmal AF. For major complications, one case of cardiac tamponade occurred in each group, both of which were resolved after pericardial puncture and drainage. For minor complications, the conventional group experienced two cases of transient phrenic nerve palsy and one case of hematoma at the puncture site, while the simplified group experienced five cases of transient phrenic nerve palsy and two cases of hematoma at the puncture site.

**Table 3:** Outcomes of Procedure

	PSM conventional group (n = 146)	PSM simplified group (n = 146)	P value
Success of first anatomical attempt	143 (97.9%)	132 (90.4%)	0.022
Acute success	145 (99.3%)	146 (100%)	0.975
Complications (major)	1 (0.7%)	1 (0.7%)	0.596
Complications (minor)	3 (2.1%)	7 (4.8%)	0.772
Follow-up (months)	11.2 ± 3.6	11.8 ± 3.8	0.329
Maintenance of sinus rhythm	114 (78.1%)	109 (74.7%)	0.842

Success of first anatomical attempt is defined as the absence of PV potentials after the first ablation circle, and no recovery of potentials is observed during the check. Acute success was defined as achievement of entrance block confirmed after the final freeze application, with no potentials recorded in any PV at the end of the procedure. PSM: propensity score matching.

### Cox regression analysis

Cox regression analysis was performed to assess the association between ablation protocol and prognosis in AF patients, and the results of Cox regression analysis are shown in Table 4. Univariable Cox regression analysis was performed for each of the following clinically relevant variables including AF type, LA diameter, course of AF, and others. In univariate Cox regression analysis, the ablation protocol was not a predictor of AF recurrence. A multivariable Cox regression model was then constructed, including all variables with P < 0.2 in univariable analysis. The results revealed that after controlling for other confounders, only non-paroxysmal AF was a predictor of AF recurrence.

**Table 4:** Analysis With Cox Regression in the Propensity Score-Matched Cohort

Variables	Univariate analysis			Multivariate analysis		
	HR	95.0% CI	P value	HR	95.0% CI	P value
Sex						
Female	1.000					
Male	0.865	0.532–1.408	0.560			
Age (years)	1.020	0.996–1.044	0.104	1.016	0.991–1.043	0.216
BMI (kg/m^2^)	1.011	0.934–1.095	0.781			
Course of AF (months)	1.002	0.999–1.005	0.232			
Classification of AF						
Paroxysmal	1.000					
Non-paroxysmal	2.243	1.390–3.619	0.001	1.859	1.057–3.271	0.031
CHA_2_DS_2_-VASc	1.082	0.875–1.337	0.469			
Basic diseases						
Hypertension	1.514	0.918–2.498	0.104	1.380	0.832–2.289	0.212
CHD	0.776	0.355–1.695	0.524			
Diabetes	1.135	0.581–2.219	0.710			
Stroke/TIA	1.243	0.569–2.716	0.585			
Heart failure	1.533	0.213–11.045	0.671			
LA diameter (mm)	1.066	1.025–1.108	0.001	1.032	0.983–1.084	0.203
Modality						
Conventional	1.000					
Simplified	1.150	0.716–1.846	0.564			

AF: atrial fibrillation; BMI: body mass index; CHD: coronary atherosclerotic heart disease; CI: confidence interval; HR: hazard ratio; LA: left atrial; TIA: transient ischemic attack.

## Discussion

This study compared the efficacy and safety of simplified temperature-guided single-freezing protocol and the conventional TTI-guided double-freezing protocol using second-generation cryoballoon in the treatment of AF. The main findings of this study are as followed. First, the temperature-guided protocol was safe and effective, and demonstrated comparable efficacy and safety to the conventional protocol. Second, simplifying the freezing protocol can significantly reduce freezing application time, freezing frequency, procedure time, and X-ray time and dose. Third, this protocol is safe and effective for both paroxysmal and persistent AF. Therefore, the simplified protocol deserves further research and clinical promotion.

Research into optimizing freezing protocols for cryoballoon ablation has progressed significantly. With the widespread application of the second-generation cryoballoon, clinical studies have demonstrated substantially improved ablation efficiency compared to the first-generation device [10], providing a practical basis for simplifying freezing operations. Growing evidence indicates that PV isolation can be achieved with high success rates using single-freezing protocols or shorter freezing durations, without compromising long-term clinical outcomes [4, 11–17]. These findings challenge the necessity of conventional double-freezing strategies and highlight the potential for safer, more efficient procedures.

Simplified cryoablation protocols currently rely on two primary observational indicators: TTI and cryoballoon temperature. TTI < 60 s is the best predictive indicator for long-term PV isolation [18–20], therefore this approach generally involves achieving TTI within 60 s followed by cryoablation for 120–180 s, without the need for a second freeze. Most studies confirm that this approach reduces procedure time and radiation exposure without compromising outcomes [21, 22]. However, PV potential may not always be observable [23, 24], complicating TTI assessment in some cases. An alternative strategy uses cryoballoon temperature as an indicator for PV isolation. Studies indicate that achieving a temperature of ≤ −40 °C within the first minute of freezing indicates good PV occlusion by the cryoballoon. Multiple studies demonstrate the safety and efficacy of temperature-only protocols which do not need TTI record [25–29]. However, these studies lack comparison with conventional freezing protocols guided by TTI. In addition, a few studies explore combined TTI/temperature parameters to optimize ablation efficacy [5, 10, 30]. However, these protocols remain under investigated, and their parameter interpretation is often complex, limiting clinical adoption.

**Figure 2:**
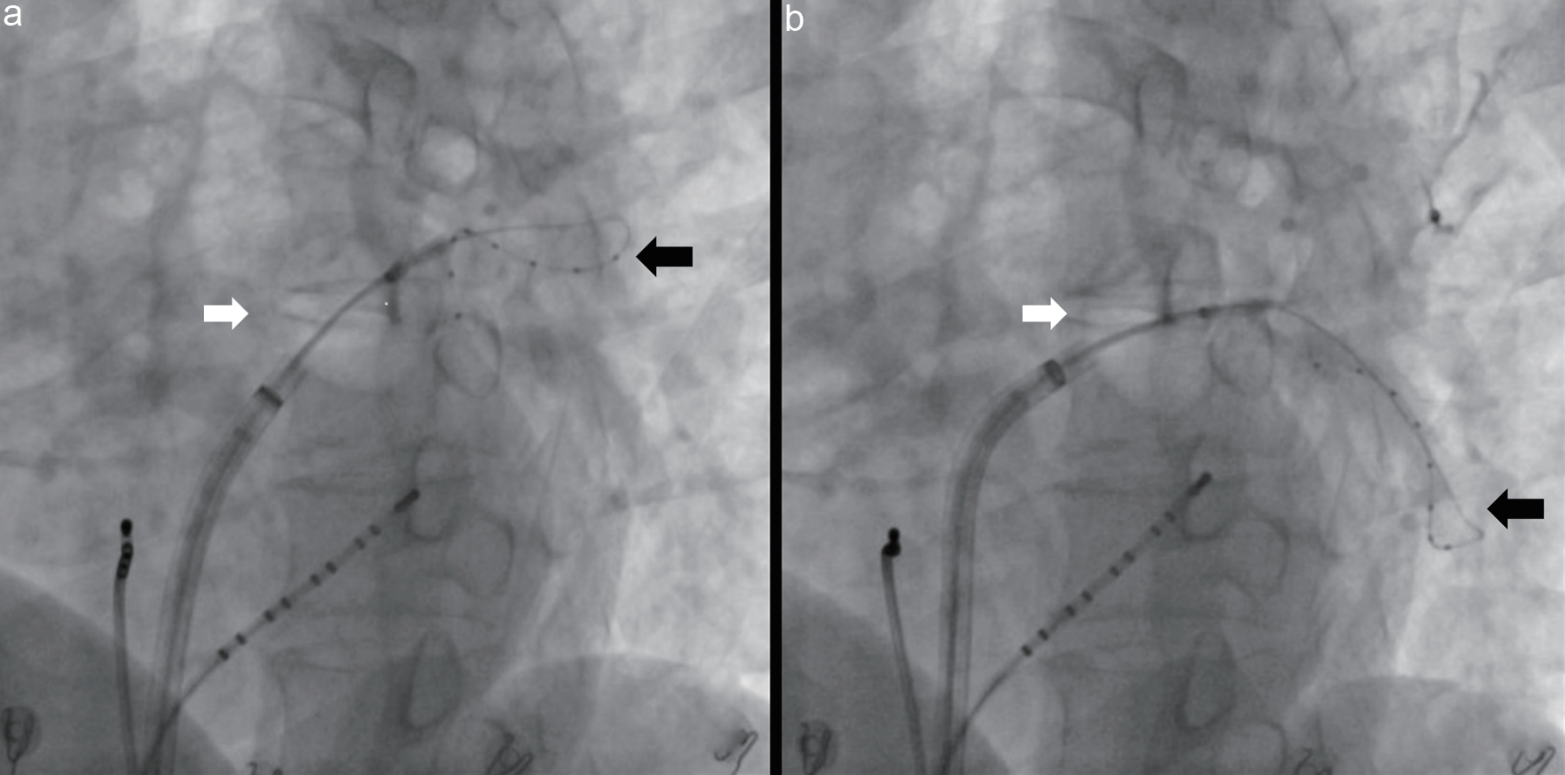
X-ray image of the relative position between the Achieve electrode (black arrow) and the cryoballoon (white arrow) during the cryoablation process. (a) The cryoballoon and Achieve electrode are both located near the pulmonary vein vestibule. (b) The cryoballoon is placed near the pulmonary vein vestibule, and the Achieve electrode penetrates deep into the pulmonary vein.

Based on our clinical experience, we have developed a simplified cryoablation protocol that relies entirely on temperature monitoring. This modified approach involves performing only a single freeze per PV. During the delivery of frozen energy, Achieve was not required to place in PV vestibule for observing potential. Instead, the Achieve was inserted deep into the PV to provide better mechanical support for maintaining optimal contact between the cryoballoon and PV vestibule (Fig. 2). The benefits of this technique are significant, as only the positions of the cryoballoon and the PV vestibule need to be considered, greatly simplifying the difficulty of the operation. The disadvantage is that no changes in PV potential can be observed during freezing. Therefore, the significantly lower first-attempt anatomical success rate observed in the simplified group is not unexpected. Although the simplified protocol omitted TTI monitoring, the definitive criterion for confirming PV isolation remains the electrophysiological documentation of absence of PV potentials. To address this limitation, two-stage verification process were implemented. First, the Achieve was used to observe PV potential prior to freezing, and record the position of Achieve that can observe PV potential under fluoroscopic guidance. After completing all PV freezing, the Achieve return to the recorded positions one by one to verify whether PV potentials have been eliminated. Any residual potentials are then targeted with supplemental freezing to ensure complete electrical isolation. It should be noted that our technique should not be interpreted as demonstrating superiority over contemporary TTI-guided approaches. It may be an alternative to TTI-guided approaches, with its value being highest in bailout situations or in resource-constrained, high-volume environments where maximizing procedural efficiency is a priority.

In terms of clinical outcomes, this simplified approach demonstrates comparable efficacy in AF treatment. There was no significant statistical difference in the acute success rate and long-term recurrence rate between the two groups. Regression analysis further confirmed that the simplified protocol was not an independent predictor of AF recurrence. The most notable advantage of this simplified protocol lies in its significant reduction of radiation exposure, especially in the freezing process. Compared with the conventional protocol, simplified protocol eliminates at least one freeze and contrast injection process per PV, greatly reducing the application of X-rays. Furthermore, the reduced procedural complexity naturally leads to lower radiation exposure, benefiting both patients and operators.

### Limitations

This study has several limitations that should be acknowledged. First, as a retrospective analysis, the findings require validation through prospective randomized controlled trials. Second, the conventional protocol in this study was based on local Chinese consensus recommendations at the time of study design. However, as the reviewer correctly noted, many contemporary cryoballoon workflows in international practice do not routinely require a bonus application when TTI is < 60 s. Therefore, the conventional arm in our study may not fully represent the most relevant contemporary benchmark. Third, a significant limitation of this study is the absence of dynamic freezing parameters such as the distribution of the time to −40 °C in each group. Consequently, we are unable to address questions pertaining to the proportion of applications reaching −40 °C within 60 s in the conventional group, or the relationship between time to −40 °C and the success rate of acute isolation. Fourth, the persistent AF subgroup had a smaller sample size, which may have limited statistical power to detect between-group differences. Finally, our follow-up protocol relied primarily on symptom-driven Holter monitoring and the follow-up period for the study is relatively short. Future studies are needed to incorporate systematic rhythm monitoring and longer-term outcome assessments to confirm the efficacy of the simplified protocol.

### Conclusion

In summary, the simplified protocol using attainment of −40 °C within the first 60 s of freezing as an indicator demonstrates comparable procedural efficacy to the conventional TTI-guided double-freeze approach. Importantly, this modified protocol achieves these equivalent clinical outcomes while significantly reducing both procedural duration and radiation exposure. While these advantages suggest that this simplified technique may be beneficial, its generalizability is limited by the single-center design and the exclusive use of second-generation balloons. Therefore, these findings should be considered preliminary, and broader clinical adoption requires validation in multicenter randomized controlled trials.

## Data Availability

The data that support the findings of this study are available on request from the corresponding author. The data are not publicly available due to privacy or ethical restrictions. Any inquiries regarding supporting data availability of this study should be directed to the corresponding author.
